# Effect of trimetazidine dihydrochloride therapy on myocardial external efficiency in pre-clinical individuals with a hypertrophic cardiomyopathy pathogenic variant: results of the ENERGY trial

**DOI:** 10.1093/cvr/cvaf120

**Published:** 2025-07-02

**Authors:** Beau Olivier van Driel, Stephan A C Schoonvelde, Sonia Borodzicz-Jazdzyk, Roy Huurman, Julia Visch, Lourens Robbers, Hans Harms, Judith Verhagen, Alexa Vermeer, Joost van den Aardweg, Albert C van Rossum, Tjeerd Germans, Michelle Michels, Jolanda van der Velden

**Affiliations:** Department of Physiology, Amsterdam Cardiovascular Sciences, Amsterdam UMC, Location VUmc, VUmc O2 building, De Boelelaan 1117, Room 11W53, 1081HV Amsterdam, The Netherlands; Department of Cardiology, Thorax Center, Cardiovascular Institute, Erasmus Medical Center, Rotterdam, The Netherlands; Department of Cardiology, Amsterdam Cardiovascular Sciences, Amsterdam UMC, Location VUmc, Amsterdam, The Netherlands; Department of Cardiology, Thorax Center, Cardiovascular Institute, Erasmus Medical Center, Rotterdam, The Netherlands; Department of Physiology, Amsterdam Cardiovascular Sciences, Amsterdam UMC, Location VUmc, VUmc O2 building, De Boelelaan 1117, Room 11W53, 1081HV Amsterdam, The Netherlands; Department of Cardiology, Amsterdam Cardiovascular Sciences, Amsterdam UMC, Location VUmc, Amsterdam, The Netherlands; MedTrace Pharma A/S, Horsholm, Denmark; Department of Clinical Genetics, Erasmus Medical Center, Rotterdam, The Netherlands; Department of Human Genetics, Amsterdam UMC, Location AMC, Amsterdam, The Netherlands; Department of Pulmonology, Amsterdam UMC, Location VUmc, Amsterdam, The Netherlands; Department of Cardiology, Amsterdam Cardiovascular Sciences, Amsterdam UMC, Location VUmc, Amsterdam, The Netherlands; Department of Cardiology, Amsterdam Cardiovascular Sciences, Amsterdam UMC, Location VUmc, Amsterdam, The Netherlands; Department of Cardiology, Thorax Center, Cardiovascular Institute, Erasmus Medical Center, Rotterdam, The Netherlands; Department of Physiology, Amsterdam Cardiovascular Sciences, Amsterdam UMC, Location VUmc, VUmc O2 building, De Boelelaan 1117, Room 11W53, 1081HV Amsterdam, The Netherlands

**Keywords:** Hypertrophic cardiomyopathy, Metabolic therapy, Trimetazidine, Randomized clinical trial, Myocardial external efficiency

## Abstract

**Aims:**

Previous studies have shown that individuals with a hypertrophic cardiomyopathy (HCM) pathogenic variant (PV) or likely pathogenic variant (LPV) without a HCM phenotype (PV/LPV carrier) have decreased myocardial external efficiency (MEE), which is thought to be a key pathomechanism in the onset and progression of HCM. Metabolic treatments improved exercise capacity in HCM patients, but evidence that such drugs correct reduced MEE is lacking. The ENERGY trial is a double-blind, placebo-controlled randomized clinical trial to define if the metabolic drug trimetazidine (TMZ) corrects reduced MEE in PV/LPV carriers for HCM.

**Methods and results:**

51 *MYBPC3* or *MYH7* PV/LPV carriers were screened after which 40 were included and randomized into a treatment group (*n* = 20) or placebo group (*n* = 20) stratified for sex. Participants were treated with TMZ 20 mg or placebo three times daily during 8 weeks. The main outcome of this study was MEE as measured by [^11^C]-acetate positron emission tomography/computed tomography (PET/CT) and cardiac magnetic resonance (CMR) scan. Secondary outcomes were exercise parameters as measured by cardio-pulmonary exercise testing (CPET). Drug safety was monitored by (serious) adverse event registration. Treatment groups were comparable in terms of age, sex, body mass index, P/LP gene variant, and echocardiographic parameters without significant differences. Baseline CMR parameters and MEE were not significantly different between treatment groups. Eight weeks of treatment with TMZ did not significantly alter MEE compared to placebo. The mean MEE changed from 30.3 ± 3.8 to 29.8 ± 4.3% in the placebo group and from 30.1 ± 4 to 29.1 ± 4% in the TMZ group. Compared to placebo, the TMZ group did not have a significantly different MEE (difference −0.44, 95% interaction CI, −2.863 to 1.986, *P* = 0.68). The mean V′O_2_max as a percentage of predicted V′O_2_max (V′O_2_max %pred) changed from 108 ± 17 to 111 ± 19 (95% CI, −6 to 10, *P* = 0.84) percent in the placebo group and from 105 ± 17 to 113 ± 14 (95% CI, 1 to 16, *P* = 0.03) percent in the TMZ group. After adjustment for baseline, the TMZ group had a significantly increased V′O_2_max %pred (difference 6.37, 95% interaction CI, −3 to 16, *P* = 0.04).

**Conclusion:**

The ENERGY trial is the first proof-of-concept randomized controlled trial to test the hypothesis that TMZ improves MEE in *MYBPC3 or MYH7* PV/LPV carriers. We conclude that metabolic therapy with TMZ may not correct the P/LP gene variant-related decrease in MEE.

**Trial registration:**

Netherlands Trial Register NL7492 (URL https://onderzoekmetmensen.nl/nl/trial/25078)

**Time of primary review: 63 days**

**See the editorial comment for this article ‘Modulating myocardial metabolism in preclinical hypertrophic cardiomyopathy pathogenic variant carriers: a window of opportunity or wishful thinking?’, by B. Raman and E. Brociek, https://doi.org/10.1093/cvr/cvaf157.**

## 1. Introduction

Hypertrophic cardiomyopathy (HCM) is the most common genetic heart disease with an estimated prevalence of 1:500 to 1:200.^1–4^ HCM is characterized by hypertrophy of the left ventricle (LV), in the absence of loading conditions. In ∼50% of HCM patients, the disease is caused by a genetic pathogenic variant (PV) or likely pathogenic variant (LPV), most frequently in a sarcomere gene. Currently >2000 P/LP HCM-related variants have been reported, of which the two most frequently affected genes (*MYH7* and *MYBPC3*) encode the sarcomere proteins myosin heavy chain (MyHC) and cardiac myosin-binding protein-C (cMyBP-C).^2,4,5^ Family screening with cascade genetic testing leads to the identification of PV/LPV carriers without an HCM phenotype.^6^ The PV/LPV carrier group provides an opportunity to define P/LP gene variant-related cardiac changes in the absence LV hypertrophy. Previous studies showed functional and anatomical abnormalities in PV/LPV carriers, such as diastolic dysfunction, myocardial crypts, myocardial ischaemia, and myocardial energy deficiency.^7–10^

Myocardial energy deficiency has been hypothesized to play a key role in HCM pathophysiology.^11^ A reduced ratio between phosphocreatine and adenosine triphosphate ATP (PCr/ATP), a measure of energetic status of the heart, was observed in PV/LPV carriers.^9^ In addition, myocardial external efficiency (MEE) was significantly lower in PV/LPV carriers of genes encoding thick filament proteins cMyBP-C and MyHC^12^ and the thin filament protein cardiac troponin T^13^ compared to healthy controls, indicating that energy deficiency is an early P/LP variant-related change in the heart before the development of LV hypertrophy. At the cardiac muscle cell level, several P/LP variant-mediated changes in myofilament function have been proposed to underlie reduced cardiac efficiency in HCM. Firstly, the P/LP variant-induced increase in ATP utilization of sarcomeres, which was reported in HCM mice.^14^ In line with these findings, studies in cardiac tissue from HCM patients showed an increased cost of muscle contraction, illustrated by a ∼2-fold higher tension cost, i.e. ratio between ATP utilization and generated force, compared to controls.^12,15^ In addition, the increase in myofilament calcium-sensitivity, which is commonly observed in *in vitro* mouse and human HCM heart muscle studies will increase both force development and ATP consumption at physiologic [Ca^2+^]. Moreover, recent studies reported a change in myosin head conformation, from the super-relaxed, low-energy, state (SRX) to a less energy-efficient disordered state (DRX) in human HCM.^16,17^ Overall, these studies show that inefficient cardiac function and altered energetic status occur at an early disease stage even before development of LV hypertrophy, and may thus represent a target for preventive therapy.

In the healthy heart, the majority of energy demand is met by oxidation of fatty acids and carbohydrates. Although fatty acids represent the predominant fuel for the heart at rest, they provide less ATP per consumed O_2_ molecule in comparison to carbohydrates.^18^ Thus, agents which shift metabolism away from the preferred fatty acids towards carbohydrates could improve ATP supply/demand balance. Metabolic agents such as trimetazidine (TMZ), which are used in clinical practice as anti-anginal agents, have been suggested to improve cardiac energetics and function.^18–20^ TMZ partially inhibits β-oxidation of fatty acids by blocking the long-chain mitochondrial 3-ketoacyl coenzyme A thiolase (3-KAT) enzyme in the mitochondria.^20,21^ Pre-clinical studies with TMZ showed that arrested isolated rat hearts immersed in cardioplegic solution with addition of TMZ have increased myocardial energetic status and functional recovery.^22,23^ A study in rat hearts exposed to global ischaemia showed that aortic-constricted hypertrophied hearts treated with TMZ had increased heart function compared to values of untreated controls.^24^ Clinical studies with TMZ have shown positive effects on exercise capacity in patients with ischaemic heart disease,^25–27^ as well as patients with heart failure but these studies lack sufficient evidence for clinical implementation.^28^ One RCT with small sample size studied TMZ in heart failure patients, and found that TMZ increased the myocardial energetic status measured by PCr/ATP ratio improved functional class and LV function.^29,30^

Abozguia *et al*. showed that metabolic therapy with perhexiline had a beneficial effect in symptomatic HCM and increased the myocardial PCr/ATP ratio.^31^ As reduced cardiac efficiency in symptomatic HCM patients is caused by both P/LP variant-mediated and secondary disease-related cardiac remodeling,^16,32^ the positive effect of metabolic therapy in HCM patients may be related to drug-mediated pleiotropic effects. There has not yet been a study which explores if metabolic therapy is able to correct the primary P/LP variant-mediated reduced cardiac efficiency. Our study measured cardiac efficiency before and after treatment with the metabolic drug TMZ in PV/LPV carriers to exclude any effects of TMZ on secondary disease-related cardiac remodelling. Specifically, we tested the hypothesis that TMZ improves MEE in PV/LPV carriers.

This clinical trial is the first proof-of-concept study to determine whether TMZ can improve MEE in PV/LPV carriers of genes encoding thick filament (*MYH7* and *MYBPC3*) proteins, as measured by state-of-the-art [^11^C]-acetate positron emission tomography/computed tomography (PET/CT) and cardiac magnetic resonance (CMR).

## 2. Methods

### 2.1 Trial design

**Figure 1:**
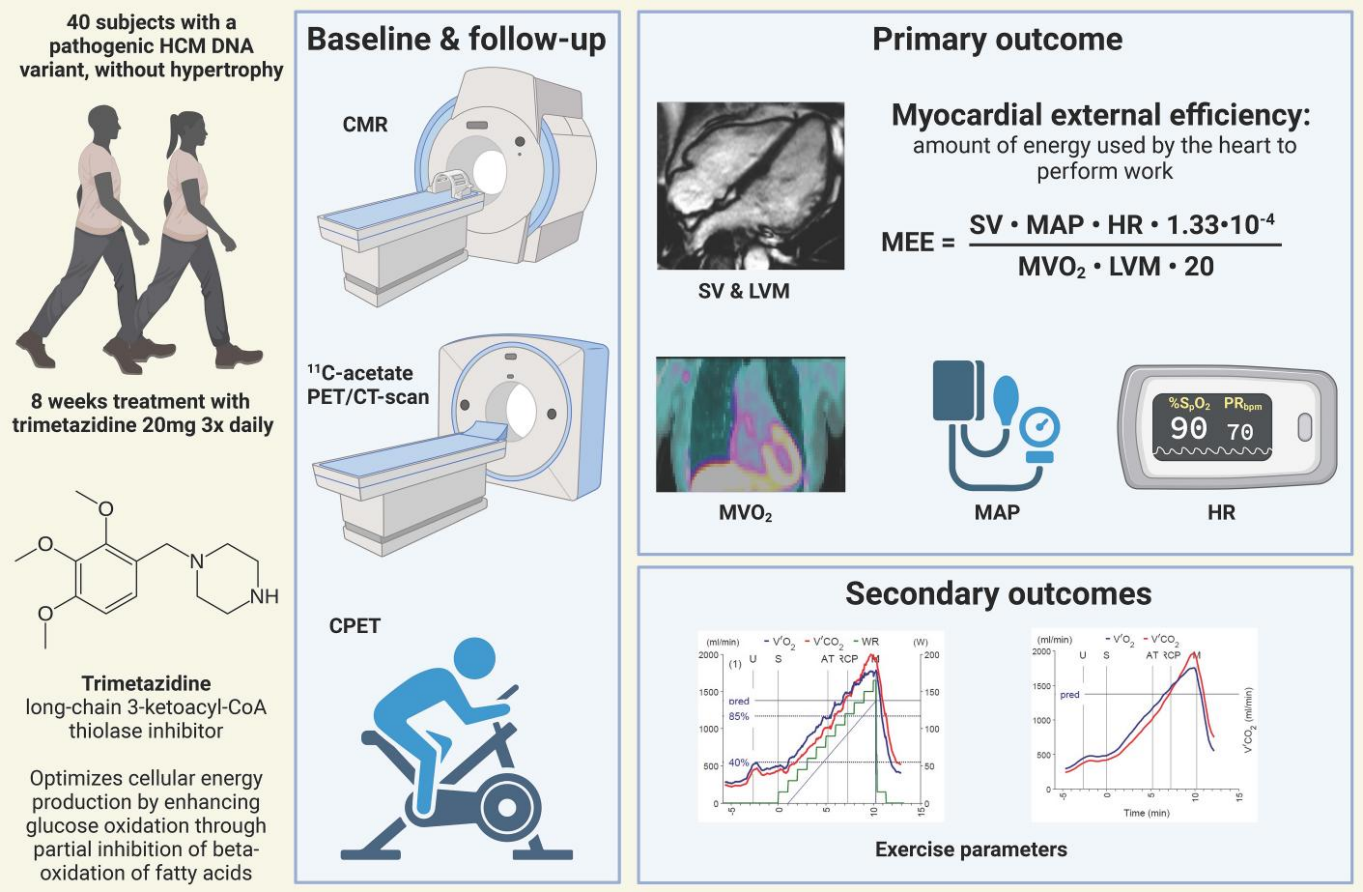
Schematic representation of the methods used in the ENERGY trial. 40 PV/LPV carriers in the *MYBPC3* or *MYH7* genes were treated with TMZ 20 mg or placebo three times daily. The primary outcome is MEE, which is calculated by dividing the amount of energy used by the heart by the amount of work it has produced. Secondary outcomes are exercise parameters, obtained by CPET. In the lower right corner, two CPET graphs are shown. The left y-axis shows gas flow in mL/min, the right axis shows work rate (WR) in watt (W) the x-axis depicts time. The blue (top), red (middle), and green (bottom) lines show the increase in oxygen flow (V′O_2_), carbon dioxide flow (V′CO_2_), and work rate (WR), respectively. Four time points are indicated by vertical lines, from left to right: U: unloaded pedaling, S: start exercise, AT: anaerobic threshold, RCP: respiratory compensation point, M: maximum work rate. HCM, hypertrophic cardiomyopathy; DNA, deoxyribonucleic acid; CMR, cardiac magnetic resonance; PET/CT, positron emission tomography/computed tomography; CPET, cardio-pulmonary exercise test; MEE, myocardial external efficiency; SV, stroke volume; LVM, left ventricular mass; MAP, mean arterial pressure; HR, heart rate; MVO_2_, myocardial oxygen consumption. Figure created in https://BioRender.com.

*Figure 1* provides an overview of the performed study procedures. The investigator-led, two centre, randomized, double-blind, placebo-controlled trial started on 13 March 2019 in the Amsterdam UMC, location VU medical centre in Amsterdam, and the Erasmus Medical Center in Rotterdam, the Netherlands. After initial start of the study with 6 participants, the study was halted for more than 2 years because of the onset of the COVID-19 pandemic. Inclusion was resumed in March 2022 and the last visit occurred on 7 March 2023.

### 2.2 Ethics

This study was conducted in accordance with good clinical practice guidelines, and in accordance with the Declaration of Helsinki. The study was approved by the Medical Ethical Testing Committee VUmc, the Central Committee on Research Involving Human Subjects (CCMO) and registered in the European Union Drug Regulating Authorities Clinical Trials Database (EudraCT). This study was also registered in the Netherlands Trial Register under Trial NL7492 (URL https://onderzoekmetmensen.nl/nl/trial/25078). All subjects provided written informed consent.

### 2.3 Study population and sample size

The high reproducibility of the methodology enables to study effects of therapy in a relatively small study group. To reveal a 15% beneficial effect of TMZ on MEE, taking into account a standard deviation of 15% based on our previous studies of MEE in HCM P/LP variant carriers,^12^ a number of 20 PV/LPV carriers were included in each group (with TMZ or placebo) to reach a power of 80% (significance level 0.05). The use of a 15% beneficial effect of TMZ on MEE in the power calculation was determined based on an increase of ATP/PCr ratio of >30% in non-obstructive HCM patients after treatment with perhexiline,^31^ and our earlier study which shows that MEE is reduced >30% in PV/LPV carriers when compared to controls.^12^ Considering that TMZ has never been used in PV/LPV carrier before, it was hypothesized that TMZ corrects half of the MEE decrease that has been observed previously.

Forty PV/LPV carriers^33^ (age 18–65 years) in *MYBPC3* and *MYH7* were recruited in the Erasmus Medical Center in Rotterdam, The Netherlands (*Table 1* and Supplementary material online, *Table S1*). After slow recruitment of PV carriers in *MYH7* (initial study protocol, as described earlier^34^) *MYBPC3* and LPV carriers for both genes were added. We chose to add only *MYBPC3* variants and no other genes to keep the patient population as homogeneous as possible with only genes encoding for thick filament proteins, with known variant-related effects on sarcomere energetics. The diagnosis of PV/LPV carrier was based on the presence of a P/LP variant and absence of LV hypertrophy, with a maximal wall thickness (MWT) of ≤14 mm on transthoracic echocardiography. The mean interventricular septum (IVS) thickness of our cohort was 9.7 mm, well below the HCM cut-off value of ≥13 mm for G+ relatives. Potential study participants were excluded if they met any of the following exclusion criteria: known cardiovascular disease, diabetes mellitus, medication use, contra-indication for CMR, inability to give informed consent, severely impaired renal function with a GFR <30 mL/min, and Parkinson disease.

**Table 1:** Baseline characteristics, per treatment arm

	Placebo (*n* = 20)	Trimetazidine (*n* = 20)
Mean age, years	44.9 ± 12.4	43.2 ± 8.8
Female, *n* (%)	13 (65)	13 (65)
Sarcomeric gene, *n* (%)		
*MYH7, n (%)*	5 (25)	3 (15)
*MYBPC3, n (%)*	15 (75)	17 (85)
Mean BMI, kg/m^2^	25.2 ± 5.6	24.5 ± 2.0
Mean BSA, m^2^	1.9 ± 0.3	1.9 ± 0.2
Systolic blood pressure, mmHg	121.6 ± 11.8	125.2 ± 20.2
Diastolic blood pressure, mmHg	72.3 ± 7.8	76.4 ± 13.5
Echocardiographic parameters		
IVS thickness, mm	9.7 ± 2.3	9.6 ± 1.9
LVPW thickness, mm	7.3 ± 0.9	7.6 ± 1.0
IVS/PW ratio	1.4 ± 0.3	1.3 ± 0.3
LVEF, %	63.3 ± 5.1	62.4 ± 6.5
LA diameter, mm	36.1 ± 4.4	35.9 ± 3.2
E: Peak early diastolic filling velocity, m/s	0.8 ± 0.1	0.7 ± 0.1
A: Peak late diastolic filling velocity, m/s	0.6 ± 0.2	0.6 ± 0.2
*e*′: early mitral annular velocity, m/s	9.8 ± 2.3	9.4 ± 2.0
*E*/*A* ratio	1.3 ± 0.3	1.3 ± 0.4
*E*/*e*′ ratio	8.1 ± 2.5	8.1 ± 1.9
Deceleration time, ms	189.5 ± 20.1	188.2 ± 25.8
LVOT flow speed, m/s	1.3 ± 0.2	1.2 ± 0.2
Presence of SAM, *n* (%)	0 (0)	0 (0)
CMR LV parameters		
EDVi, mL/m^2^	76.1 ± 10.7	80 ± 10.6
ESVi, mL/m^2^	28.5 ± 5.4	29.9 ± 1.5
SVi, mL/m^2^	47.6 ± 8.3	50.1 ± 6.3
LVMi, g/m^2^	45.5 ± 7.7	48.2 ± 4.6
LVEF, %	63.7 ± 1.1	62.0 ± 1.1
MEE parameters		
External work, mmHg/mL	7643 ± 1571	8013 ± 1615
Heart rate, bpm	59 ± 8.9	59 ± 6.9
MVO_2_, ml O_2_/g/min	0.11 ± 0.02	0.11 ± 0.02
LVM, g	88.9 ± 22.4	93.3 ± 20.0
MEE, %	30.3 ± 3.8	30.1 ± 4
CPET parameters		
V′O_2_max, mL/min/kg	30.2 ± 7.3	28.9 ± 7.1
V′O_2_max, % of predicted	108 ± 17	105 ± 17
Maximum work rate, W	221 ± 62	213 ± 55
Respiratory exchange ratio	1.2 ± 0.1	1.2 ± 0.1
V′E/V′CO_2_	30.4 ± 3.9	29.5 ± 2.7

MYH7, gene encoding myosin heavy chain; MYBPC3, gene encoding myosin binding protein C; BMI, body mass index; BSA, body surface area; IVS, interventricular septum; LVPW, left ventricular posterior wall; LVEF, left ventricular ejection fraction; LA, left atrium; LVOT, left ventricular outflow tract; SAM, systolic anterior motion; EDVi, end-diastolic volume indexed for BSA; ESVi, end-systolic volume indexed for BSA; SVi, stroke volume indexed for BSA; LVMi, LV mass indexed for BSA; MEE, myocardial external efficiency; MVO_2_, myocardial oxygen consumption; CPET, cardio-pulmonary exercise test; V′O_2_max, maximal oxygen consumption; V′E/VCO_2_, ventilatory efficiency expressed as the fraction of exhaled CO_2_ of total ventilation. Mean +/−SD.

### 2.4 Clinical evaluation

Clinical evaluation included medical history, physical examination, ECG, and transthoracic echocardiography. Standard 12-lead ECG was performed in the supine position during quiet respiration. LV hypertrophy was evaluated with the Romhilt–Estes criteria. Pathological Q waves were defined as duration > 40 ms or depth > 30% R wave in ≥ 2 contiguous leads. T-wave inversion was defined as ≥ 3 mm in ≥ 2 leads. Echocardiographic studies were analysed according to the European Society of Cardiology guidelines.^35,36^ MWT, left atrial size, leaflet, and chordal systolic anterior motion of the mitral valve, and resting LV outflow tract peak velocity were assessed.^35^ LV diastolic function was defined as normal, abnormal relaxation, pseudonormal, or restrictive filling, based on Doppler mitral inflow pattern parameters including early (*E*) and late (*A*) LV filling velocities, *E*/*A* ratio, and tissue Doppler imaging-derived septal early diastolic velocities (*e*′).^37^

### 2.5 Genetic testing and variant classification

All recruited individuals carried P/LP variants (see Supplementary material online, *Table S1*).^38^

### 2.6 Randomization, blinding, and treatment allocation

Study participants were randomly assigned to treatment 1:1 placebo/TMZ and stratified for sex. A validated variable block randomization model was used, which is a randomization algorithm constructed in such a way that randomized inclusions are divided across groups (with stratification for sex) in variable block sizes. This is done to ensure true randomness during the allocation.^39^ Study participants and investigators were blinded for the assigned treatment through electronic data capture software Castor EDC.^40^ Only after measurement of all outcome assessments were investigators unblinded for the assigned treatment.

### 2.7 Preparation and encapsulation of study medication

The investigational medicinal product and matching placebo were manufactured under GMP conditions by the Amsterdam UMC. Verum capsules consisted of a TMZ dihydrochloride 20 mg tablet (Vastarel®, Les laboratoires Servier, PA0568/033/001 or Idaptan®, Danval, S.A, Spain, 56.704), and microcrystalline cellulose. The matching placebo consisted of capsules with only microcrystalline cellulose. The capsules were packed in flasks and labelled according to GMP Annex 13.

### 2.8 Treatment of study participants

Study participants were treated with TMZ 20 mg or placebo three times daily during 8 weeks.

### 2.9 CMR acquisition and analysis

CMR scans were performed at a 3.0 Tesla whole body MR scanner (MAGNETOM Vida, Siemens Healthcare, Erlangen, Germany). The protocol consisted of cine imaging, native T1 mapping, 2D flow mapping for aortaflow quantification, and late gadolinium enhancement after administration of gadolinium-based contrast agent (godoterate meglumine, Dotarem, Guerbet, Paris, France) into the antecubital vein at 0,4 mL/kg. Blood pressure measurements were performed in the MRI scanner, before the start of the scanning protocol and shortly before infusing of the contrast agent. CMR images were analysed using commercially available software (CVI42, Circle Cardiovascular Imaging, Calgary, Canada). LV volume analysis was performed by contouring the endocardial contours in end-diastole and end-systole in a stack of short axis slices covering the whole LV, with inclusion of the papillary muscles in the LV volume.

### 2.10 [^11^C]-acetate positron emission tomography

A detailed description of the methods used can be found in the supplement. In short, after a fasting period over >4 h, PET scans were performed on a Philips Ingenuity TF PET/CT scanner. Following a scout CT scan, a low-dose CT scan was performed. After this, a 50-min list mode emission scan was performed, starting simultaneously with automated injection of 378 ± 37 MBq [^11^C]acetate as a 5–10 mL bolus (1 mL/s) in a peripheral vein, followed by a 35-mL saline flush (2.0 mL/s). Blood pressure measurements were performed in the PET scanner, before the start of the scanning protocol and during 5-min intervals for 15 min after injection of [^11^C]acetate. PET scans were analysed using aQuant,^41^ available at no cost for collaborative, non-commercial research purposes via https://medtracepharma.com/aquant/.

### 2.11 Calculation of MEE

MEE is the amount of oxygen consumed by the heart to perform work. [^11^C]-acetate PET/CT imaging was used to indirectly quantify myocardial oxygen consumption (MVO_2_). CMR was performed to calculate cardiac mechanical external work (EW), which is the product of stroke volume and mean arterial pressure. With MVO_2_ and EW, MEE can be calculated using the following equation:


$$\mathrm{MEE}=\frac{\mathrm{SV}\times \mathrm{MAP}\times \mathrm{HR}\times 1,33\;\times 10^{-4}}{{\mathrm{MVO}}_{2}\times \mathrm{LVM}\times 20}$$

MEE = Myocardial external efficiency, SV = stroke volume, MAP = mean arterial pressure, HR = heart rate, and LVM = left ventricular mass.

### 2.12 Cardio-pulmonary exercise test

Cardio-pulmonary exercise tests (CPET) were performed according to the incremental exercise protocol described in the joint statement on CPET by the American Thoracic Society and the American College of Chest Physicians.^42^ The protocol consists of symptom-limited incremental exercise, with 3 min rest, 3 min unloaded pedaling, followed by incremental exercise with steps of x W/min, where x is chosen as an integer multiple of 5, so that the expected maximum is attained after ∼10 min. Reference values were used from the population based Study of Health in Pomerania.^43^

### 2.13 Data capture and statistical analysis

Data were captured using Castor EDC.^40^ Data were exported into GraphPad Prism (GraphPad Prism version 9.3.1 for Windows, GraphPad Software, San Diego, California USA, www.graphpad.com) for statistical analysis and generation of figures for presentation of study data.

Primary and secondary study parameters are all quantitative, continuous variables. Variables are summarized by means ± SD or median and inter-quartile range, depending on whether they are normally distributed or not. Normality was assessed using normal-probability plots. The primary analysis is based on intention-to-treat. Methods were used, such as mixed model analyses that are valid under the assumption that data are missing at random. This mixed model uses a compound symmetry covariance matrix, and is fit using restricted maximum likelihood. In the absence of missing values, this method gives the same *P*-values and multiple comparisons tests as repeated measures ANOVA. In the presence of missing values (missing completely at random), the results can be interpreted like repeated measures ANOVA. In the current study, values were missing for random logistical reasons related to planning schedules of the CPET facilities, leading to six missing CPET’s: two in the baseline placebo group, two in the baseline TMZ group, and two in the follow-up placebo group. No participant missed both CPET’s. There were no missing values for the primary study parameter MEE.

Multiple comparisons were corrected using statistical hypothesis testing with the Šídák method for the secondary study parameters. Results are considered statistically significant with a two-tailed *P*-value < 0.05.

The primary study parameter MEE was analysed with an ANOVA analysis. A linear regression model was used with measurements on follow-up as the dependent variable and treatment group (TMZ or placebo group) and the baseline measurement as independent variables. The secondary study parameters were analysed in a mixed model analysis. The models include treatment group, time point, and interaction as fixed independent variables. The models also include a random effect for subjects. Residual analyses showed robustness and reliability of the model.

## 3. Results

### 3.1 Recruitment

**Figure 2:**
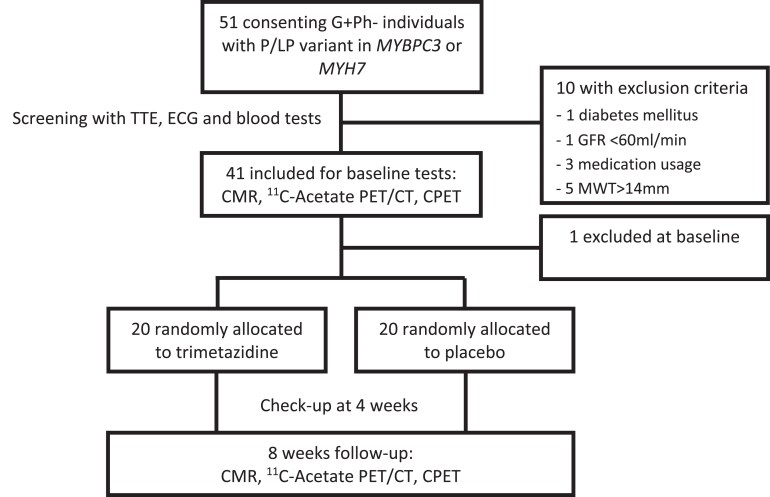
Flowchart of study procedures and inclusion of study participants. No study participants dropped out during the medication phase of the study. PV/LPV, pathogenic variant/likely pathogenic variant; *MYBPC3*, myosin binding protein C3; *MYH7*, myosin heavy chain 7; TTE, transthoracic echocardiography; ECG, electrocardiography; CMR, cardiac magnetic resonance; PET/CT, positron emission tomography/computed tomography; CPET, cardio-pulmonary exercise test.

Participants were recruited at the cardiomyopathy outpatient clinic in the Erasmus Medical Center Rotterdam, the Netherlands between 13 March 2019 and 14 December 2022. 51 potential participants were screened, of whom 41 were included into the study and 10 who had exclusion criteria (specifically diabetes de novo, medication use, renal insufficiency with GFR <60 mL/min, and five participants had MWT >14 mm). *Figure 2* summarizes participant flow during the study. After inclusion, one participant was excluded before start of treatment because of an incidental finding of perimyocarditis on the baseline CMR scan. Of 40 participants randomized, 20 were in the placebo group and 20 were in the TMZ group. No participants dropped out during the treatment phase of the study.

### 3.2 Baseline characteristics

Participant demographics and clinical variables are shown in *Table 1*. The placebo and TMZ groups were comparable, as can be seen from similar mean ± SD for, respectively, age: 45 ± 12.4 vs. 43.2 ± 8.8 years, percentage female: 65% vs. 65%, and body mass index: 25.2 ± 5.6 vs. 24.5 ± 2.0 kg/m^2^. Three participants had a MWT of 13 or 14 mm. One participant met the ECG Romhilt–Estes LVH criteria, and MWT on echocardiography was 12 mm. Average MWT was 9.7 ± 2.3 mm in the placebo group and 9.6 ± 1.9 mm in the TMZ group. Additional echocardiographic parameters are shown in *Table 1* and baseline LV parameters measured by CMR are shown in Supplementary material online, *Figure S1*.

### 3.3 Treatment with placebo or TMZ

40 Participants were treated with placebo or TMZ for an average of 57 ± 6 days. Based on pill counts, overall therapy adherence was excellent at 94.4 ± 6.2%, and therapy adherence for the TMZ group was 92.2 ± 8.6%. Participants had no self-reported side-effects of treatment, but upon inquiry 12 participants (4 in placebo and 8 in TMZ group) reported mild and transient symptoms of nausea, abdominal pain, softer stool, and/or dizziness only during the first week of treatment. No serious adverse events or suspected unexpected serious adverse reactions occurred during the study.

### 3.4 Parameters for calculation of MEE

**Figure 3:**
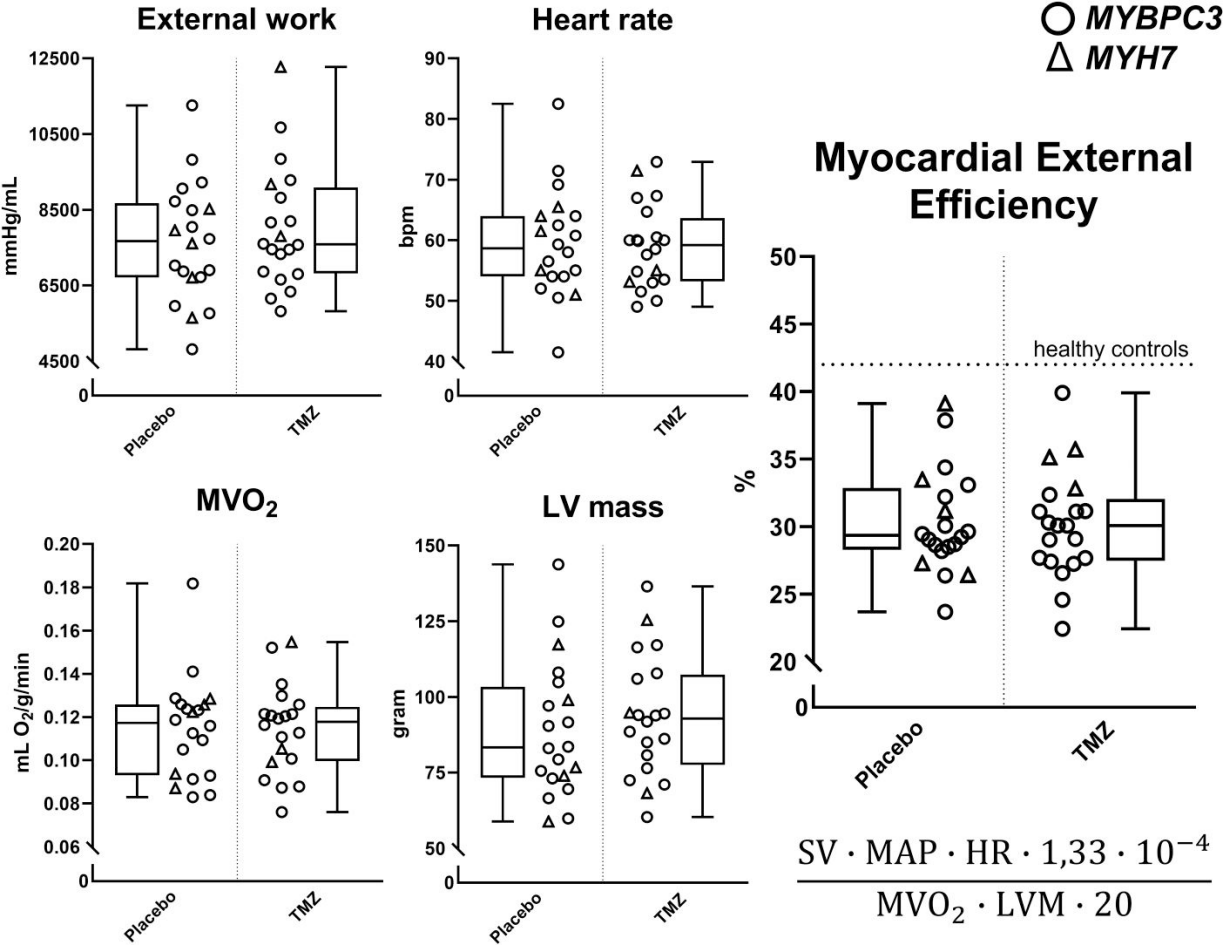
Baseline parameters for MEE calculation. Each graph contains a scatterplot accompanied by a Tukey boxplot, which depict EW, HR, MVO_2_, LVM, and MEE in the placebo (*n* = 20) and TMZ (*n* = 20) groups. Subjects with a P/LP variant in the *MYBPC3* and *MYH7* gene are depicted by circles and triangles, respectively. The dotted horizontal line shows mean MEE of healthy controls, as reported previously.^12^

The parameters for MEE calculation are EW, HR, MVO_2_, and LVM and are shown in *Figure 3*. Mean EW was 7643 ± 1571 vs. 8013 ± 1615 mmHg/mL, HR 59 ± 8.9 vs. 59 ± 6.9 bpm, MVO_2_ 0.11 ± 0.02 vs. 0.11 ± 0.02 mL O_2_/g/min, LVM 88.9 ± 22.4 vs. 93.3 ± 20.0 g in the placebo vs. TMZ group, respectively. MEE was 30.3 ± 3.8% in the placebo group and 30.1 ± 4% in the TMZ group. There were no statistically significant differences in any of the parameters and MEE between the placebo and TMZ groups before treatment. Moreover, MEE values at baseline were comparable to earlier data from PV/LPV carriers and lower compared to healthy controls in our previous study.^12^ Lastly, MEE values at baseline were significantly higher in participants <40 years old (*n* = 12) compared to participants >40 years old (*n* = 28), namely 32.6 ± 4.6 vs. 29.2 ± 3% (95% CI, −5.9 to −0.9, *P* = 0.008) (see Supplementary material online, *Figure S2*).

### 3.5 Effect of TMZ on MEE

**Figure 4:**
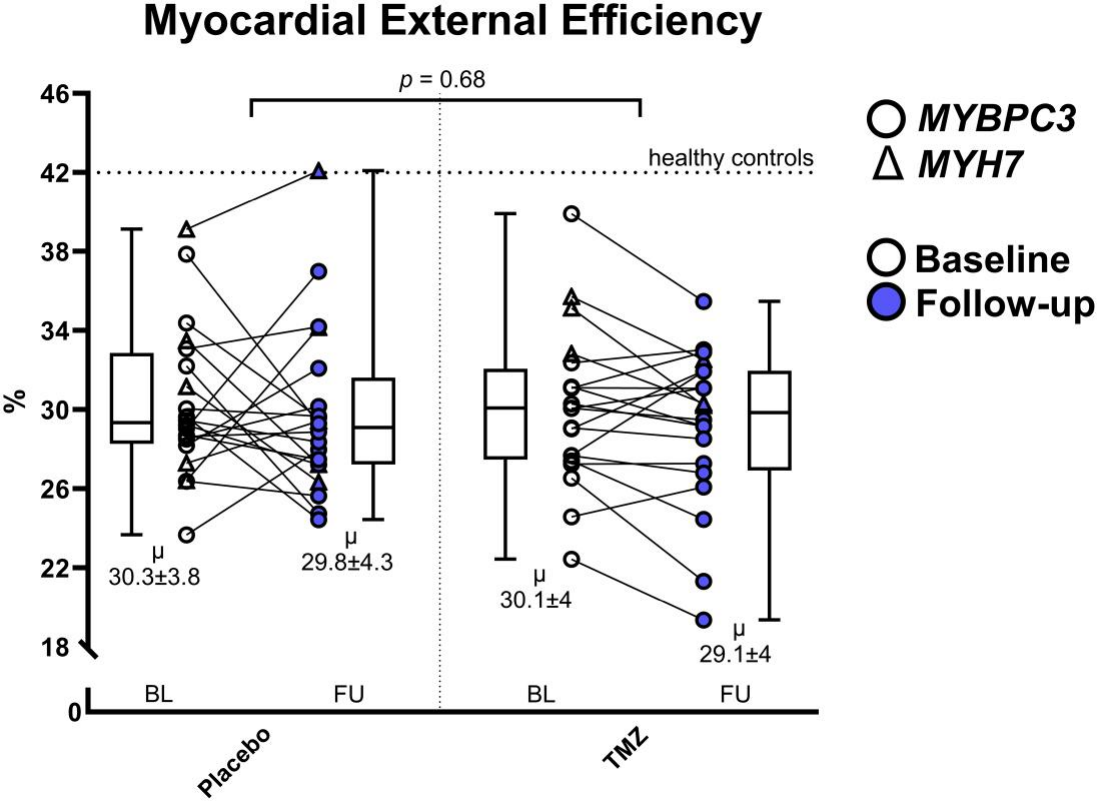
Baseline and follow-up myocardial external efficiency in the placebo (*n* = 20) and TMZ (*n* = 20) groups. Baseline and follow-up values of individual subjects are connected by a line. Tukey boxplots show median and spread, with numerical values for mean and standard deviation below. Subjects with a P/LP variant in the *MYBPC3* and *MYH7* gene are depicted by circles and triangles, respectively. Baseline values are white, follow-up values are blue. The dotted horizontal line shows mean MEE of healthy controls, as reported previously.^12^ Statistical significance was determined by mixed model analysis.

Eight weeks of treatment with TMZ did not significantly alter MEE compared to placebo. MEE changed from 30.3 ± 3.8 to 29.8 ± 4.3 (95% CI, −2.5 to 1.5, *P* = 0.79) percent in the placebo group and from 30.1 ± 4 to 29.1 ± 4 (95% CI, −2.9 to 1.0, *P* = 0.46) percent in the TMZ group. After adjustment for baseline, the TMZ group did not have a significantly altered MEE (difference −0.44, 95% interaction CI, −2.863 to 1.986, *P* = 0.68). *Figure 4* shows the individual changes in MEE. In a subgroup analysis, after adjustment for baseline, the TMZ group did not have a significantly altered MEE in participants <40 years old (*n* = 6, difference −0.55, 95% interaction CI, −6.7 to 5.6, *P* = 0.95) or in participants >40 years (*n* = 12, difference 0.86, 95% interaction CI −1.8 to 3.5, *P* = 0.6). It is important to note that these subgroup analyses are inadequately powered. Supplementary material online, *Figure S2* shows the individual changes in MEE in the subgroup analysis.

### 3.6 Effect of TMZ on exercise parameters

**Figure 5:**
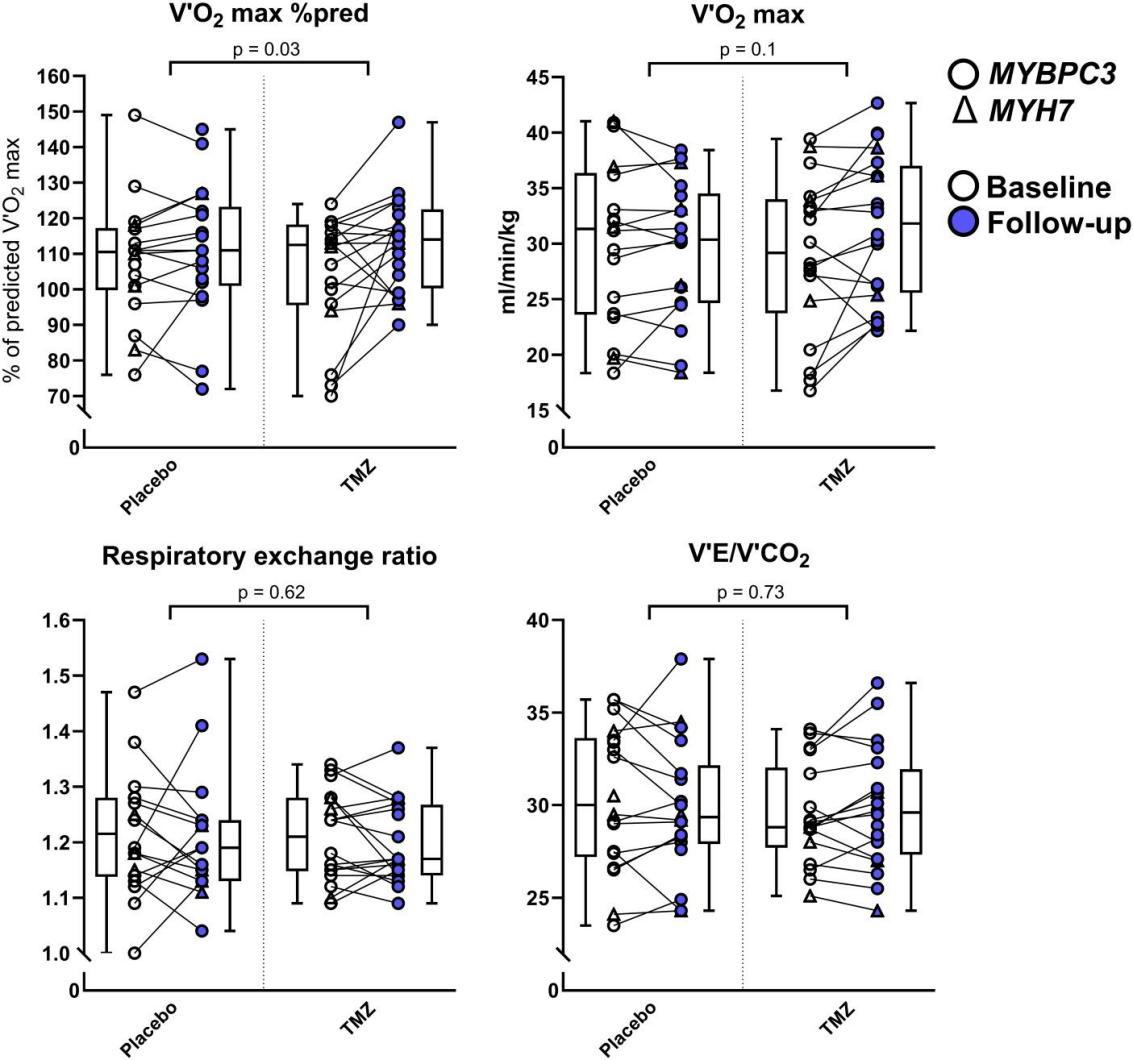
Baseline and follow-up V′O_2_ max as percentage of predicted V′O_2_ max (top left), V′O_2_ max corrected for bodyweight (top right), respiratory exchange ratio (bottom left), and V′E to V′CO_2_ (bottom right) in the placebo (*n* = 20) and TMZ (*n* = 20) groups. Baseline and follow-up values of individual subjects are connected by a line. Tukey boxplots show median and spread. Subjects with a P/LP variant in the *MYBPC3* and *MYH7* gene are depicted by circles and triangles, respectively. Baseline values are white, follow-up values are blue. Statistical significance was determined by mixed model analysis, multiple comparisons were corrected using statistical hypothesis testing with the Šídák method.

The mean V′O_2_max as a percentage of predicted V′O_2_max (V′O_2_max %pred) changed from 108 ± 17 to 111 ± 19 (95% CI, −6 to 10, *P* = 0.84) percent in the placebo group and from 105 ± 17 to 113 ± 14 (95% CI, 1 to 16, *P* = 0.03) percent in the TMZ group. After adjustment for baseline, the TMZ group had a significantly increased V′O_2_max %pred (difference 6.37, 95% interaction CI, −3 to 16, *P* = 0.04). The mean V′O_2_max/kg changed from 30.2 ± 7.3 to 29.6 ± 6.2 mL/min/kg (95% CI, −2.1 to 1.9, *P* = 0.99) in the placebo group and from 28.9 ± 7.1 to 31.5 ± 6.5 mL/min/kg (95% CI, 0.2 to 4, *P* = 0.03) in the TMZ group. After adjustment for baseline, the TMZ group showed a trend towards significance for an increased V′O_2_max/kg (difference 2.14, 95% interaction CI, −0.289 to 4.567, *P* = 0.1). The mean RER changed from 1.217 ± 0.11 to 1.210 ± 0.11 (95% CI, −0.046 to 0.044, *P* = 0.99) in the placebo group and from 1.210 ± 0.08 to 1.197 ± 0.07 (95% CI, −0.056 to 0.030, *P* = 0.74) in the TMZ group. After adjustment for baseline, the TMZ group did not have a significantly altered RER (difference −0.012, 95% interaction CI, −0.067 to 0.042, *P* = 0.62). The mean V′E/V′CO_2_ changed from 30.4 ± 3.9 to 29.8 ± 3.6 (95% CI, −1.32 to 0.82, *P* = 0.83) in the placebo group and from 29.4 ± 2.7 to 29.8 ± 3.2 (95% CI, −0.55 to 1.49, *P* = 0.49) in the TMZ group. After adjustment for baseline, the TMZ group did not have a significantly altered V′E/V′CO_2_ (difference 0.72, 95% interaction CI, −0.565 to 2.0, *P* = 0.73). *Figure 5* shows the individual changes in V′O_2_max %pred, V′O_2_max/kg, RER, and V′E/V′CO_2_. The additional exercise parameters time to max work, work rate at anaerobic threshold, maximum work rate, O_2_pulse, V′O_2_ during unloaded pedaling, V′O_2_ at anaerobic threshold, and ΔV′O_2_/ΔWR are shown in Supplementary material online, *Figure S3*. TMZ did not alter any of these parameters.

## 4. Discussion

Based on this study in PV/LPV carriers, we conclude that the metabolic drug TMZ may not correct the primary P/LP variant-related decrease in MEE observed in this study and our earlier studies.^12,13,44^ Unlike most anti-anginal drugs that decrease MVO_2_ by unloading the heart through negative inotropy, TMZ is an anti-anginal drug that does not alter the hemodynamic state of the body. Its cardioprotective effect is not yet fully understood, but it has been suggested to improve the metabolic state of the heart through a number of mechanisms, including: improvement of energy efficiency by inhibition of free fatty acid β-oxidation, inhibition of fibrosis, and reduction of oxidative stress, calcium overload, and acidosis in the cardiomyocyte.^20,45–47^ Studies in patients with coronary artery disease and ischaemic cardiomyopathy show beneficial effects of TMZ on metabolic markers for vascular function, inflammation, and cardiac fibrosis as well as clinical outcomes such as number of weekly angina attacks, exercise tolerance and major acute cardiovascular events.^48–50^ Although secondary disease-related pathomechanisms such as fibrosis, oxidative stress, and myocardial ischaemia play a major role in HCM and may precede clinical progression of HCM by years,^10,51^ our current study found no effect of TMZ treatment on MEE.

Other clinical studies on modulation of substrate metabolism in HCM are limited to three RCT’s. The first study was performed by Abozguia *et al*. in 46 symptomatic non-obstructive HCM patients treated with perhexiline 100 mg or placebo. Perhexiline increased myocardial energetic status, measured by ^31^P-magnetic resonance spectroscopy, improved exercise capacity and improved New York Heart Association class.^31^ Perhexiline is a partial inhibitor of carnitine palmitoyl transferase and, like TMZ, is suggested to shift substrate metabolism from preferred fatty acids to glucose oxidation, which yields more ATP for the same amount of O_2_ consumption.^52^ There are concerns of toxic side effects of perhexiline, mainly hepatitis and peripheral neuropathy, which necessitates careful therapeutic drug monitoring.^53^ The RESOLVE-HCM study was announced in 2021 and will further explore the effects of perhexiline in HCM.^54^ The second study was performed by Coats *et al*., who concluded that TMZ 20 mg three times daily had no effect on exercise capacity in symptomatic non-obstructive HCM patients.^55^ The third and most recent study, performed by Maron *et al*., showed that ninerafaxstat, a novel 3-KAT inhibitor, improves exercise performance of non-obstructive HCM patients, especially those most symptomatically limited.^56^

Our study showed a minor effect of TMZ on exercise capacity compared to the placebo-treated group (*Figure 5*), which is in line with the effects of metabolic drugs on exercise capacity in two of three studies mentioned in the previous paragraph. However, these studies have a population consisting of mostly symptomatic HCM patients taking medication and some treated by septal myectomy or alcohol septum ablation. This study population is markedly different from our study population of otherwise healthy PV/LPV carriers, which is shown by great differences in exercise capacity (V′O_2_max 50–60% of predicted compared to 105–108% in our study). A study by Verwijs *et al*. even hypothesized a beneficial effect of sarcomeric gene variants on physical performance before the onset of cardiomyopathy.^57^ Comparisons should therefore be made with caution, especially since our study was not powered for secondary outcomes from exercise testing. Moreover, the study by Coats *et al*., which is most comparable to ours, was underpowered as it included only 49 participants of the intended 72 for adequate statistical power.^55^ Despite this, our observed increase in V′O_2_max after treatment with TMZ is striking and exceeds that of cardiac myosin inhibitors (CMI’s) which have proven effective in multiple clinical trials in obstructive HCM.^58,59^ Perhaps an explanation can be sought in the fact that TMZ’s mechanism of action includes skeletal muscle whereas CMI’s are limited to cardiac muscle. On a side note, a recent case study demonstrated beneficial effects of CMI aficamten on myocardial perfusion and energetics measured by CMR in a patient with obstructive HCM.^60^ Lastly, the abovementioned RCTs in non-obstructive HCM patients^31,55,56^ involved a mix of genotype-positive (G+) and genotype-negative (G−) individuals. Our recent study in BMI-matched obstructive HCM patients revealed diverse metabolic remodelling in cardiac tissue from G+ compared to G− individuals.^61^ The latter indicates that effects of drugs targeting cardiac energetics may differ between G+ and G− individuals. Thus, genotype should be taken into account in future RCTs.

### 4.1 Limitations

Our composite endpoint of MEE consists of multiple parameters that each have their own spread and standard deviation. The estimated SD we used for the calculation of sample size and statistical power for this study was based on previous clinical studies with single MEE measurements per participant. Consecutive MEE measurements within a relatively short time period had not yet been performed in this study population, and in the current study, we see significant spread in both treatment groups. It is possible that the cumulative spread of the MEE parameters, mainly differences in HR and blood pressure, is larger than anticipated, which may mask a potential, though small, effect of TMZ.

It is possible that a longer treatment phase with TMZ would be necessary to detect a difference in MEE during follow-up. However, the largest clinical trials with TMZ, conducted in patients with ischaemic heart disease, showed positive effects on exercise tolerance after a treatment period of 8–12 weeks.^25–27^ Given the limited clinical evidence of TMZ on MEE, we treated with TMZ for 8 weeks to minimize participant burden, based on earlier positive results after 8 weeks of treatment.

Our cohort was relatively old, and as such the likelihood of being lifelong non-penetrant may be high. However, our aim was not to study the effect of TMZ on disease onset and progression, but rather to establish the effect of TMZ on the reduced MEE that is observed in these PV/LPV carriers.

Translational perspective This study is an effort to translate extensive pre-clinical research on cardiomyocyte function and energetics in HCM into clinical practice. Based on our study in a well-defined PV/LPV carrier cohort, and previous RCTs in patients with manifest HCM, we conclude that metabolic drugs may not correct primary P/LP variant-mediated defects in cardiac energetics, but rather exert a positive effect on secondary disease-related pathomechanisms.

## 5. Conclusion

Based on our study in a well-defined PV/LPV carrier cohort, and previous RCTs in patients with manifest HCM, we conclude that metabolic drugs such as TMZ may not correct primary P/LP variant-mediated defects in cardiac energetics, but rather exert a positive effect on secondary disease-related pathomechanisms.

## Supplementary Material

cvaf120_Supplementary_Data

## Data Availability

Data available on request.
