# Supplementary material for: Effect of trimetazidine dihydrochloride therapy on myocardial external efficiency in pre-clinical individuals with a hypertrophic cardiomyopathy pathogenic variant: results of the ENERGY trial
Source: Cardiovasc Res. 2025 Jul 2;121(12):1917–28. doi: 10.1093/cvr/cvaf120 (PMC12551387; doi:10.1093/cvr/cvaf120)
Supplement: cvaf120_Supplementary_Data [file cvaf120_supplementary_data.zip › Supplement to ENERGY trial manuscript.docx]

**Supplement**

**Methods**

**[^11^C]Acetate positron emission tomography**

[^11^C]Acetate was synthesized according to Kruijer et al. [1], using a slightly modified procedure and a home-build radiosynthesis module [2]. Briefly, cyclotron produced [^11^C]CO_2_ (IBA Cyclone 18/9, Louvain-la-Neuve, Belgium, approximately 25 GBq at end of bombardment) was bubbled into a solution of 0.3 mL of methyl magnesium chloride in tetrahydrofuran, freshly made from 0.1 mL of 3M methyl magnesium chloride in tetrahydrofuran (Sigma-Alldrich, Zwijndrecht, The Netherlands) and 1.2 mL of dry tetrahydrofuran (Sigma-Alldrich). The obtained [^11^C]acetate was purified over 2 solid phase extraction columns, first a Ag+ column to extract the remaining chloride ions and second an OH- column to trap [^11^C]acetate. After washing the OH-column with water for injection, the product was eluted from the OH- column with 10 mL of a freshly prepared sterile and pyrogen free acetate/citric acid buffer (140 mg of sodium acetate trihydrate, 320 mg of sodium citrate dehydrate and 160 mg of citric acid monohydrate in 20 mL of water for injection). The final solution of [^11^C]Acetate in acetate/citrate buffer was transferred to a sterile vial via a sterile 0.22 µm filter (Millex GV), yielding a sterile, pyrogen free solution of 1900-5600 MBq of [^11^C]acetate with a (radio)chemical purity of > 98% at the end of synthesis. HPLC analysis was performed on a Dionex LPG-3400RS UPLC (Thermo Fisher, Landsmeer, Nederland), equipped with on-line GABI radiodetection (Elysia, Liege, Belgium) using a BioRad Aminex ion exclusion HPX-87H column (300x7.8 mm) with 1 µM H_2_SO_4_ solution as eluent at 0.6 mL/min. The product was detected at 229 nm.

[^11^C]Acetate PET imaging was performed to indirectly quantify oxygen metabolism using the rate constant K_2_. K_2_ represents the rate of transfer of radioactivity from tissue to blood, of which myocardial oxygen consumption (MVO_2_) can be derived as described previously [3]. After a fasting period over >4 hours, PET scans were performed on a Philips Ingenuity TF PET/CT scanner. Following a scout CT scan, a low-dose CT scan was performed. After this, a 50-minute list mode emission scan was performed, starting simultaneously with automated injection of 378 ± 37 MBq [^11^C]acetate as a 5–10 mL bolus (1 mL/s) in a peripheral vein, followed by a 35-mL saline flush (2.0 mL/s). Blood pressure measurements were performed in the PET scanner, before the start of the scanning protocol and during 5-minute intervals for 15 minutes after injection of [^11^C]acetate. PET scans were analyzed using aQuant [4] (available at no cost for collaborative, non-commercial research purposes via https://aquantsoft.com/go/aquantresearch)

1. Kruijer, P.S., et al., *A Practical Method for the Preparation of [C-11] Acetate.* Applied Radiation and Isotopes, 1995. **46**(5): p. 317-321.

2. Windhorst, A.D., et al., *A complete, multipurpose, low cost, fully automated and GMP compliant radiosynthesis system.* Journal of Labelled Compounds and Radiopharmaceuticals, 2001. **44**(S1): p. S1052-S1054.

3. Sun, K.T., et al., *Simultaneous measurement of myocardial oxygen consumption and blood flow using [1-carbon-11]acetate.* J Nucl Med, 1998. **39**(2): p. 272-80.

4. Harms, H.J., et al., *Automatic generation of absolute myocardial blood flow images using [15O]H2O and a clinical PET/CT scanner.* Eur J Nucl Med Mol Imaging, 2011. **38**(5): p. 930-9.

**Figure Legends**

**Figure S1:** Baseline LV end-diastolic volume, LV systolic volume, LV stroke volume, and LV mass in the placebo (n=20) and TMZ (n=20) groups, from top left to bottom right, respectively. Tukey boxplots show median and spread. Subjects with a P/LP variant in the *MYBPC3* and *MYH7* gene are depicted by circles and triangles, respectively.

**Figure S2:** Top: Baseline myocardial external efficiency in participants <40 years old (n=12) and participants >40 years old (n=28).

Bottom left: baseline and follow-up myocardial external efficiency in participants <40 years old in the placebo (n=6) and TMZ (n=6) groups. Bottom right: baseline and follow-up myocardial external efficiency in participants >40 years old in the placebo (n=14) and TMZ (n=14) groups.

Baseline and follow-up values of individual subjects are connected by a line. Tukey boxplots show median and spread, with numerical values for mean and standard deviation below. Subjects with a P/LP variant in the *MYBPC3* and *MYH7* gene are depicted by circles and triangles, respectively. Baseline values are white, follow-up values are blue. The dotted horizontal line shows mean MEE of healthy controls, as reported previously [12]. Statistical significance was determined by mixed model analysis.

**Figure S3:** Baseline and follow-up work rate at anaerobic threshold, maximum work rate, time to max work, VO_2_ during unloaded pedaling, O_2_ pulse, and delta VO_2_/delta work rate in the placebo (n=20) and TMZ (n=20) groups, from top left to bottom right, respectively. Baseline and follow-up values of individual subjects are connected by a line. Tukey boxplots show median and spread. Subjects with a P/LP variant in the *MYBPC3* and *MYH7* gene are depicted by circles and triangles, respectively. Baseline values are white, follow-up values are blue. Statistical significance was determined by mixed model analysis, multiple comparisons were corrected using statistical hypothesis testing with the Šídák method.
