# Supplementary material for: Effect of trimetazidine dihydrochloride therapy on myocardial external efficiency in pre-clinical individuals with a hypertrophic cardiomyopathy pathogenic variant: results of the ENERGY trial
Source: Cardiovasc Res. 2025 Jul 2;121(12):1917–28. doi: 10.1093/cvr/cvaf120 (PMC12551387; doi:10.1093/cvr/cvaf120)
Supplement: cvaf120_Supplementary_Data [file cvaf120_supplementary_data.zip › Supplementary Figures ENERGY trial.pptx]

## Slide 1
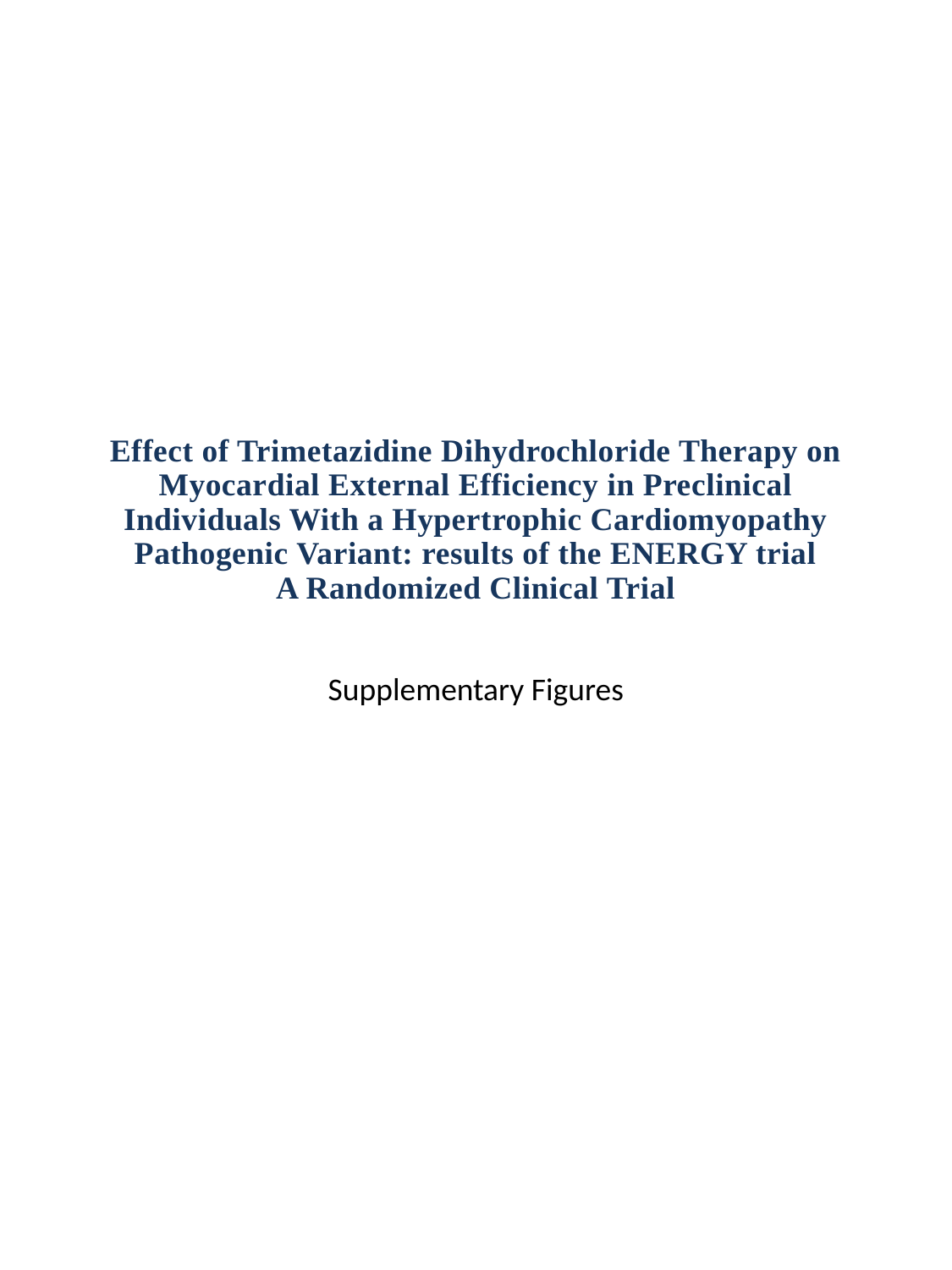

# Effect of Trimetazidine Dihydrochloride Therapy on Myocardial External Efficiency in Preclinical Individuals With a Hypertrophic Cardiomyopathy Pathogenic Variant: results of the ENERGY trial A Randomized Clinical Trial
Supplementary Figures

## Slide 2
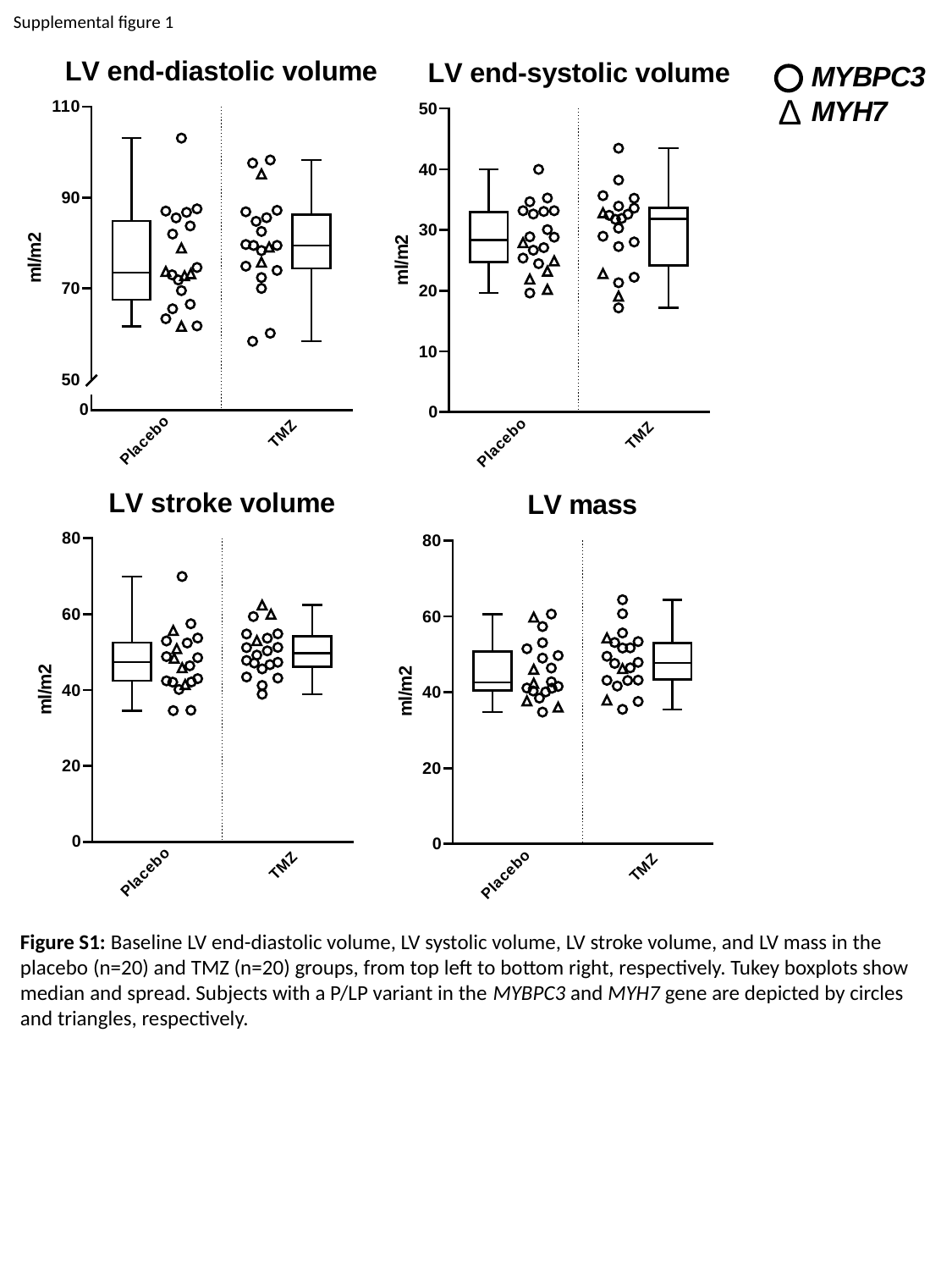

Supplemental figure 1
Figure S1: Baseline LV end-diastolic volume, LV systolic volume, LV stroke volume, and LV mass in the placebo (n=20) and TMZ (n=20) groups, from top left to bottom right, respectively. Tukey boxplots show median and spread. Subjects with a P/LP variant in the MYBPC3 and MYH7 gene are depicted by circles and triangles, respectively.

## Slide 3
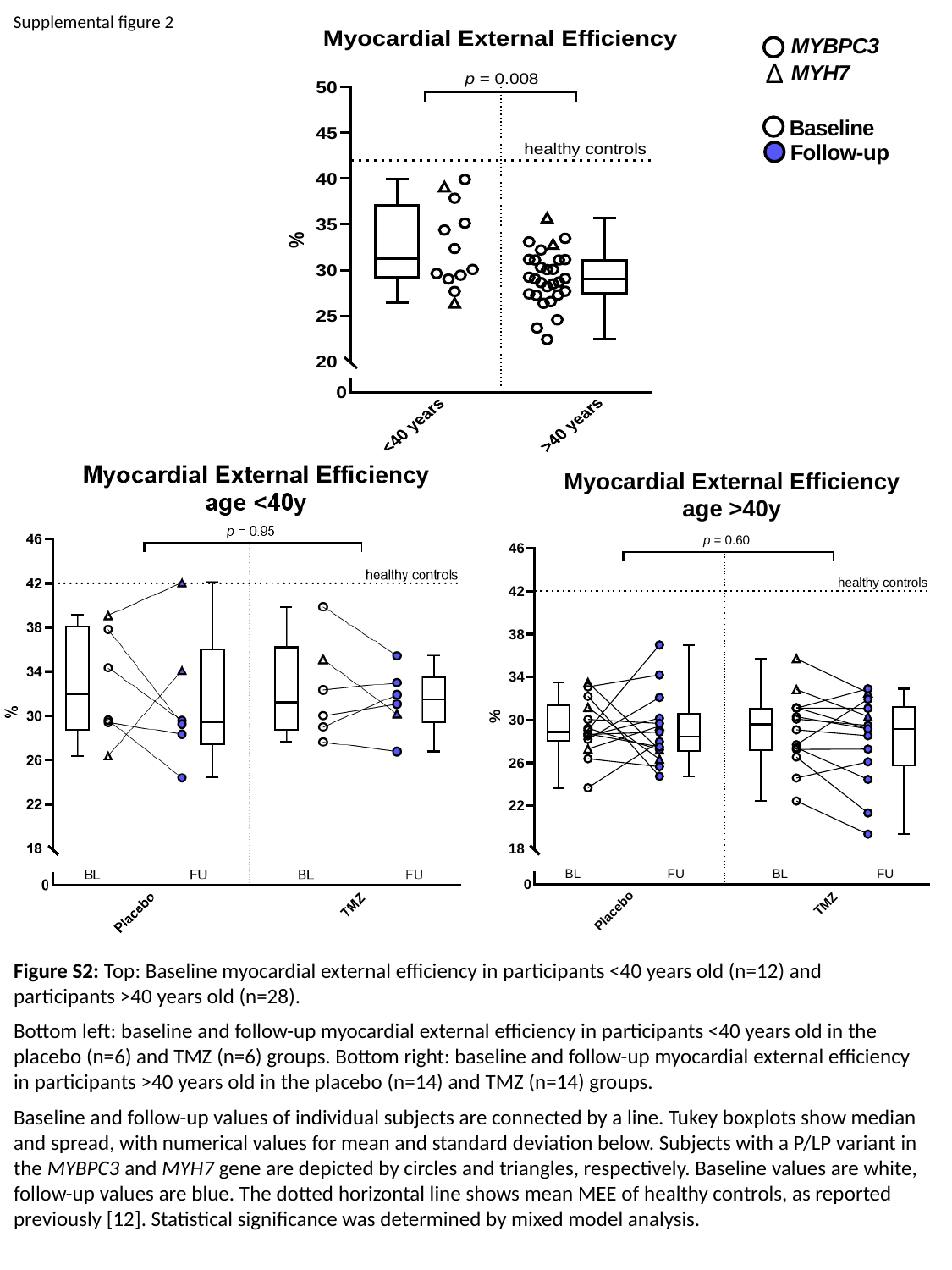

Supplemental figure 2
Figure S2: Top: Baseline myocardial external efficiency in participants <40 years old (n=12) and participants >40 years old (n=28).
Bottom left: baseline and follow-up myocardial external efficiency in participants <40 years old in the placebo (n=6) and TMZ (n=6) groups. Bottom right: baseline and follow-up myocardial external efficiency in participants >40 years old in the placebo (n=14) and TMZ (n=14) groups.
Baseline and follow-up values of individual subjects are connected by a line. Tukey boxplots show median and spread, with numerical values for mean and standard deviation below. Subjects with a P/LP variant in the MYBPC3 and MYH7 gene are depicted by circles and triangles, respectively. Baseline values are white, follow-up values are blue. The dotted horizontal line shows mean MEE of healthy controls, as reported previously [12]. Statistical significance was determined by mixed model analysis.

## Slide 4
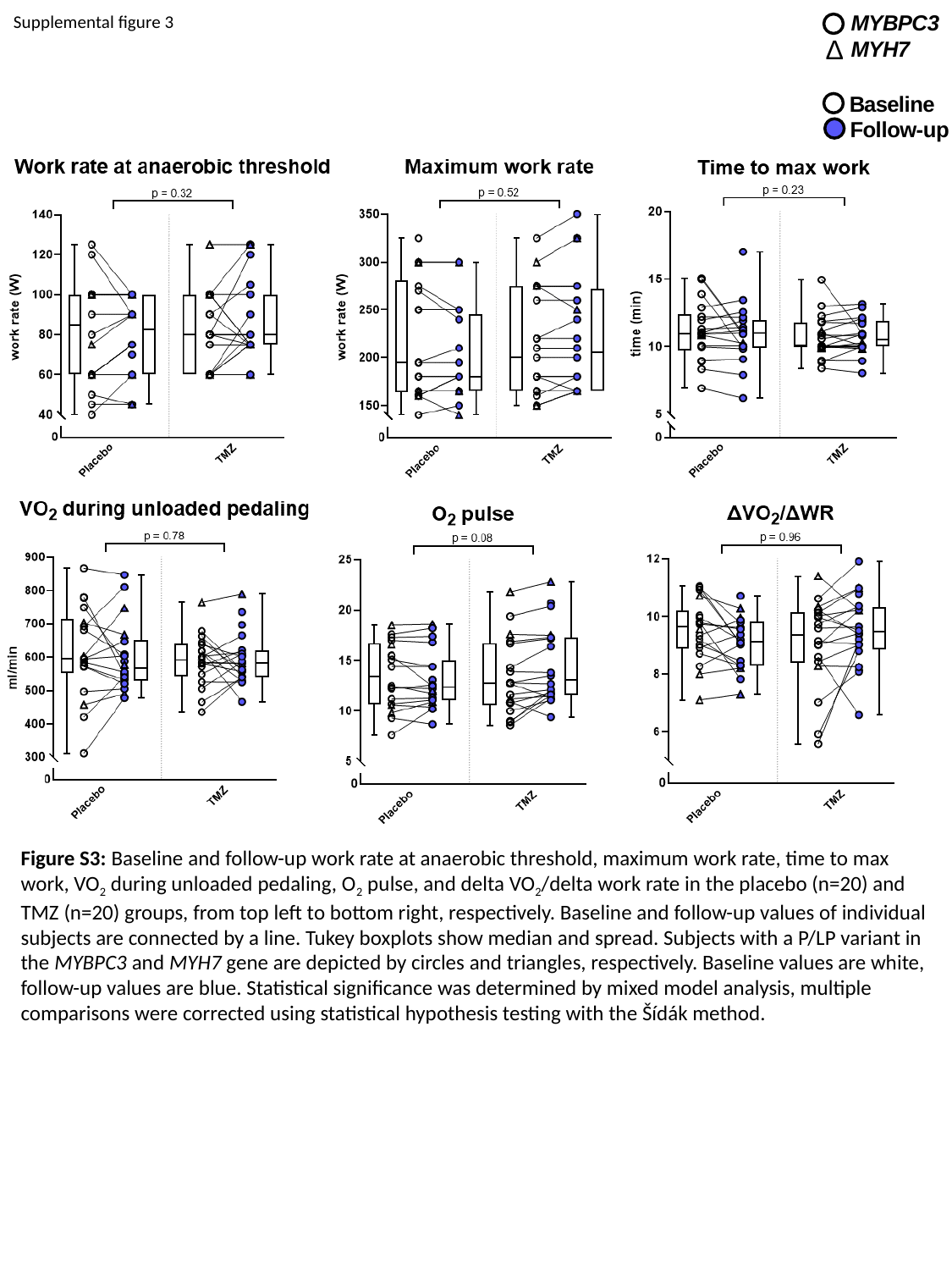

Supplemental figure 3
Figure S3: Baseline and follow-up work rate at anaerobic threshold, maximum work rate, time to max work, VO2 during unloaded pedaling, O2 pulse, and delta VO2/delta work rate in the placebo (n=20) and TMZ (n=20) groups, from top left to bottom right, respectively. Baseline and follow-up values of individual subjects are connected by a line. Tukey boxplots show median and spread. Subjects with a P/LP variant in the MYBPC3 and MYH7 gene are depicted by circles and triangles, respectively. Baseline values are white, follow-up values are blue. Statistical significance was determined by mixed model analysis, multiple comparisons were corrected using statistical hypothesis testing with the Šídák method.
