# Supplementary material for: Effect of trimetazidine dihydrochloride therapy on myocardial external efficiency in pre-clinical individuals with a hypertrophic cardiomyopathy pathogenic variant: results of the ENERGY trial
Source: Cardiovasc Res. 2025 Jul 2;121(12):1917–28. doi: 10.1093/cvr/cvaf120 (PMC12551387; doi:10.1093/cvr/cvaf120)
Supplement: cvaf120_Supplementary_Data [file cvaf120_supplementary_data.zip › Supplementary Table.docx]

| Individual | Affected gene | Gene variant | Pathogenicity |
| --- | --- | --- | --- |
| *TMZ group* |  |  |  |
| 1 | MYH7 | c.5135G>A | P |
| 2 | MYH7 | c.5786C>T | LP |
| 3 | MYBPC3 | c.2373insG | P |
| 4 | MYBPC3 | c.2373insG | P |
| 5 | *MYBPC3* | c.2373insG | P |
| 6 | *MYH7* | c.5135G>A | P |
| 7 | *MYBPC3* | c.2373insG | P |
| 8 | *MYBPC3* | c.2827C>T | P |
| 9 | *MYBPC3* | c.1458-6G>A | P/LP |
| 10 | *MYBPC3* | c.654+1G>A | P |
| 11 | *MYBPC3* | c.932C>A | P |
| 12 | *MYBPC3* | c.2373insG | P |
| 13 | *MYBPC3* | c.2827C>T | P |
| 14 | *MYBPC3* | c.2373insG | P |
| 15 | *MYBPC3* | c.2373insG | P |
| 16 | *MYBPC3* | c.927-2A>G | P |
| 17 | *MYBPC3* | c.2373insG | P |
| 18 | *MYBPC3* | c.442G>A | P/LP |
| 19 | *MYBPC3* | c.2827C>T | P |
| 20 | *MYBPC3* | c.1458-6G>A | P/LP |
| *Placebo group* |  |  |  |
| 1 | *MYH7* | c.4130C>T | P |
| 2 | *MYH7* | c.4130C>T | P |
| 3 | *MYH7* | c.1816G>A | P/LP |
| 4 | *MYH7* | c.4130C>T | P |
| 5 | *MYBPC3* | c.2373insG | P |
| 6 | *MYBPC3* | c.2373insG | P |
| 7 | *MYBPC3* | c.2373insG | P |
| 8 | *MYBPC3* | c.2938C>T | LP |
| 9 | *MYBPC3* | c.2827C>T | P |
| 10 | *MYBPC3* | c.654+1G>A | P |
| 11 | *MYBPC3* | c.2373insG | P |
| 12 | *MYBPC3* | c.932C>A | P |
| 13 | *MYBPC3* | c.2827C>T | P |
| 14 | *MYBPC3* | c.3776delA | P/LP |
| 15 | *MYH7* | c.5135G>A | P |
| 16 | *MYBPC3* | c.2373insG | P |
| 17 | *MYBPC3* | c.2827C>T | P |
| 18 | *MYBPC3* | c.1831G>A | P/LP |
| 19 | *MYBPC3* | c.2373insG | P |
| 20 | *MYBPC3* | c.2827C>T | P |

***Table S1.*** *Genotype of individual participants of this study*
