# Supplementary figures and images for: Effect of trimetazidine dihydrochloride therapy on myocardial external efficiency in pre-clinical individuals with a hypertrophic cardiomyopathy pathogenic variant: results of the ENERGY trial
Source: Cardiovasc Res. 2025 Jul 2;121(12):1917–28. doi: 10.1093/cvr/cvaf120 (PMC12551387; doi:10.1093/cvr/cvaf120)

## CONSORT 2010 Flow Diagram

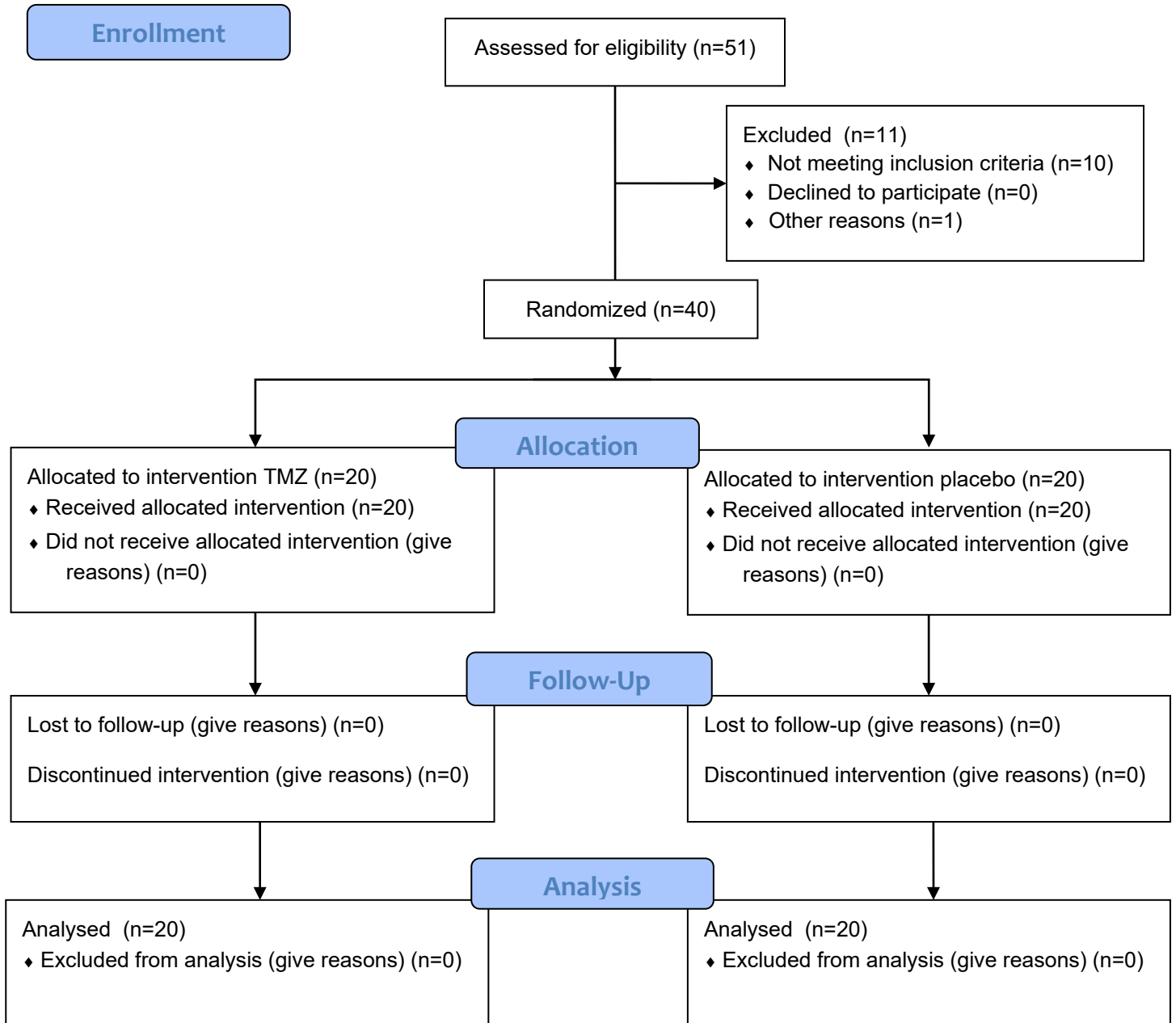

Supplement: cvaf120_Supplementary_Data [file cvaf120_supplementary_data.zip › CONSORT 2010 Flow Diagram.pdf]
